# Variations in 5S rDNAs in diploid and tetraploid offspring of red crucian carp × common carp

**DOI:** 10.1186/s12863-017-0542-2

**Published:** 2017-08-08

**Authors:** Lihai Ye, Chun Zhang, Xiaojun Tang, Yiyi Chen, Shaojun Liu

**Affiliations:** State Key Laboratory of Developmental Biology of Freshwater Fish, College of Life Sciences, Hunan Normal University, Changsha, 410081 China

**Keywords:** Diploid hybrid fish, Allotetraploid fish, 5S rDNA, Evolution, Gene conversion

## Abstract

**Background:**

The allotetraploid hybrid fish (4nAT) that was created in a previous study through an intergeneric cross between red crucian carp (*Carassius auratus* red var., ♀) and common carp (*Cyprinus carpio* L., ♂) provided an excellent platform to investigate the effect of hybridization and polyploidization on the evolution of 5S rDNA. The 5S rDNAs of paternal common carp were made up of a coding sequence (CDS) and a non-transcribed spacer (NTS) unit, and while the 5S rDNAs of maternal red crucian carp contained a CDS and a NTS unit, they also contained a variable number of interposed regions (IPRs). The CDSs of the 5S rDNAs in both parental fishes were conserved, while their NTS units seemed to have been subjected to rapid evolution.

**Results:**

The diploid hybrid 2nF_1_ inherited all the types of 5S rDNAs in both progenitors and there were no signs of homeologous recombination in the 5S rDNAs of 2nF_1_ by sequencing of PCR products. We obtained two segments of 5S rDNA with a total length of 16,457 bp from allotetraploid offspring 4nAT through bacterial artificial chromosome (BAC) sequencing. Using this sequence together with the 5S rDNA sequences amplified from the genomic DNA of 4nAT, we deduced that the 5S rDNAs of 4nAT might be inherited from the maternal progenitor red crucian carp. Additionally, the IPRs in the 5S rDNAs of 4nAT contained A-repeats and TA-repeats, which was not the case for the IPRs in the 5S rDNAs of 2nF_1_. We also detected two signals of a 200-bp fragment of 5S rDNA in the chromosomes of parental progenitors and hybrid progenies by fluorescence in situ hybridization (FISH).

**Conclusions:**

We deduced that during the evolution of 5S rDNAs in different ploidy hybrid fishes, interlocus gene conversion events and tandem repeat insertion events might occurred in the process of polyploidization. This study provided new insights into the relationship among the evolution of 5S rDNAs, hybridization and polyploidization, which were significant in clarifying the genome evolution of polyploid fish.

## Background

Most teleosts have undergone a teleost-specific genome duplication and cyprinids have emerged as the most economically important teleost family [1]. Red crucian carp (*Carassius auratus* red var., 2n = 100) and common carp (*Cyprinus carpio* L., 2n = 100) belong to two different genera of cyprinid, and bisexual fertile allotetraploid fish (4nAT) are the intergeneric hybrid between them [2]. The first two generations of hybrids were not tetraploid fishes. The tetraploid hybrids came from the fertilization of unreduced diploid sperms and eggs generated by diploid 2nF_2_. Then, the males and females of allotetraploid 4nF_3_ hybrids generated diploid sperm and diploid eggs, which were fertilized to form the allotetraploid 4nF_4_, thereby producing a bisexual fertile tetraploid fish population. Thus, two processes were involved in forming the allotetraploid fish population, first hybridization then polyploidization. In vertebrates, unlike in plants, polyploidy tends to be lethal. Therefore, the successful development of the allotetraploid fish was significant, and provided a new model to understand the evolution of allopolyploid animals.

The 5S rDNAs in higher eukaryotes are tandemly arrayed and composed of a 120-bp conserved coding sequence (CDS) and variable non-transcribed spacer (NTS) units [3], and play critical roles in ribosome folding and functionality. Data from molecular analysis studies demonstrate the existence of 5S rDNA variants [4, 5] within individuals and species of fungi [6], plants [7], and animals [8]. The long-term evolution of these variants may be mediated by a mixed mechanism involving birth-and-death and concerted evolution [9], and the occurrence rates varied in different genera. Because of the special structure of the 5S rDNA multigene, 5S rDNA is a good marker for a better understanding of the variability, organization, and evolution of fish species [10, 11]. In most eukaryotes, the 5S rDNAs are detected in distinct areas of the genome, organized as one or more tandemly repeated clusters, and their chromosomal locations can be detected by fluorescence in situ hybridization (FISH). In some cases, 5S rDNAs are interspersed with other multicopy genes, such as histones, 45S rDNAs, and repeated trans-spliced leader sequences [12]. However, the multiple localizations of 5S rDNAs and their non-colocalization with the major ribosomal gene [13], nucleolus organizer regions, and sex chromosomes are commonly found in fishes [14]. Further, the chromosomal locations of 5S rDNAs may vary among polyploid hybrids and their parental species [15], which make 5S rDNAs useful in analyzing the hereditary relationships among them.

Almost all previous molecular analyses of 5S rDNAs were based on PCRs, which unambiguously detected genetic mutations in the CDS and NTS regions of 5S rDNAs, but failed to reveal their array characteristics in genomes. To address this problem, we considered that bacterial artificial chromosome (BAC) clones inserted with large genomic DNA segments may provide a good solution. We used blood taken from 4nF_22_ to construct a BAC library of allotetraploid fish [16], and some of the materials and data from that study have been used in the present study. While it is clear that the allotetraploids witnessed the fusion of two different genomes of both progenitors, knowledge of the changes that occurred in the genomes of the allotetraploid hybrids in the evolutionary process over more than 20 generations is limited. Further, the kind of impact that hybridization and polyploidization may have had on the allotetraploid genes is also unknown. In the present study, we aimed to provide some insights into the genetic and 5S rDNA evolution of allotetraploid fish. First, we conducted a detailed structural analysis of 5S rDNAs in diploid and tetraploid hybrids of red crucian carp × common carp to determine if the evolution of 5S rDNAs involved a conversion event. Second, we carried out a comparative analysis of the 5S rDNAs among diploid and tetraploid hybrid fishes and their parental species to decipher the genetic evolution of 5S rDNAs in hybrid progenies.

## Methods

### PCR amplification and sequencing of 5S rDNAs

The fish samples in this study were collected from the Engineering Center of Polyploid Fish Breeding of National Education Ministry located at Hunan Normal University, China. The PCR templates were extracted from genomic DNA (extracted from blood) of red crucian carp (RCC), common carp (CC), and their diploid hybrid 2nF_1_ and tetraploid hybrid 4nF_22_ (ten individuals in each of the four sample groups). Fish blood was collected with sterile injectors. To minimize the suffering we caused to fish, narcotic drugs was fed before blood sampling. One pair of primers (5′-TATGCCCGATCTCGTCTGATC-3′ and 5′-CAGGTTGGTATGGCCGTAAG C-3′) [17] was synthesized by Sangon Biotech (Shanghai) Co., Ltd. The PCR cycles were as follows: 94 °C for 5 min; 30 cycles of denaturation at 94 °C for 30 s, annealing at 56 °C for 30 s, and elongation at 72 °C for 1 min; and last extension at 72 °C for 10 min. Mixtures of the obtained PCR fragments were cloned using *Escherichia coli* DH5α. A total number of 160 positive clones (40 clones for each type of fish) were tested by PCR and then sequenced by Nanjing Genscript. Homologous 5S rDNA sequences were aligned using Clustal W software (version 1.7).

### Access to 5S rDNA sequences in the 4nAT BAC library

We aligned the amplified genomic and BAC full-length 5S rDNA sequences to obtain better results, because the lengths of the sequences gained by PCR were limited. One hundred BAC clones were picked randomly from the 4nAT BAC library for BAC-ends sequencing by Nanjing Genscript. The BAC-ends sequencing was performed on ABI 3730 xl machines and the BAC-end sequences were analyzed by PHRED software. The vector sequences in the BAC-end sequences were cut out with the NCBI-VecScreen tool (https://www.ncbi.nlm.nih.gov/tools/vecscreen) and the BAC-end sequences were identified by Blastx searches, using an E-value threshold of 1e − 10. After the BAC-end sequences were annotated, 5S rDNA segments were detected in BAC clone 4nAT-150B4, which was then picked for full-length sequencing. The sequencing and assembly of the 4nAT-150B4 BAC clone was performed using Illumina next-generation sequencing technology and PacBio RS platform. Thereafter, the 5S rDNA sequence in the 4nAT-150B4 BAC (BAC-type 5S) was marked by RepeatMasker (http://www.repeatmasker.org/).

### Identification of heterozygosity of 4nAT

To determine if the 4nAT individuals we analyzed contain chromosomic regions from both parental lines, red crucian carp and common carp, we compared the BAC full length sequence 4nAT-150B4 with the genomes of both RCC and CC by local Blastn.

### Comparison of 5S rDNAs of hybrid fishes and parental species

To identify all the types of 5S rDNA repeats in the parental progenitors, the homologous 5S rDNA sequences (listed in Table 1) were compared with the genomes of both parental progenitors by local Blastn alignments. Whole-genome sequencing of the maternal RCC was completed in our laboratory together with Yunnan University, China. The genomic data are unpublished (http://rd.biocloud.org.cn/). The genome sequence of the paternal CC was downloaded from the NCBI genome website (http://www.ncbi.nlm.nih.gov/genome/?term=common%20carp). Because the 5S rDNA genes are composed of conserved CDSs and variable NTSs, we aligned only the 82-bp NTS regions in the 5S rDNA sequences of RCC, CC, and their hybrids progeny 2nF_1_ and 4nAT.

**Table 1:** The 5S rDNA sequences used to compare with genomes of both progenitors

Fish Species	RCC	CC	2nF_1_	4nF_22_
GenBank numbers	KM359663	KM359667	KM359672, KM359673	KM359675, KM359679

### Fluorescence in situ hybridization with a 200-bp probe

The chromosomal locations of the 5S rDNAs of diploid hybrid 2nF_1_, tetraploid hybrid 4nF_22_, and their diploid progenitors were analyzed by FISH. Chromosome preparations were carried out as described previously [18]. FISH was performed according to the method described by Masaru and Hideo with minor modifications [17]. The FISH probe was a 200-bp 5S rDNA repeat sequence amplified from the genomic DNA of the paternal progenitor and labeled with Dig-11-dUTP using a PCR DIG Probe Synthesis Kit (Roche, Germany).

## Results

### 5S rDNA of 4nAT detected in a BAC sequence

**Fig. 1:**
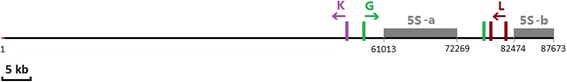
Two 5S rDNA segments detected in the BAC clone AT150B4 sequence. K: uncharacterized protein K02A2.6-like *Xenopus* (Silurana) *tropicalis*; G: G2/M phase-specific E3 ubiquitin-protein ligase-like isoform X2 (*Danio rerio*); L: uncharacterized protein LOC101883163 (*Danio rerio*). The numbers in the figure correspond to base positions of the BAC AT150B4 sequence. The *arrows* indicate the transcriptional direction of the three genes. The last exon of K gene was not detected in the BAC sequence

The BAC-ends sequencing of 100 4nAT BAC clones produced 176 BAC-end sequences, and the 5S rDNA was identified in BAC clone AT150B4 (GenBank accession number: KJ424358). The BAC AT150B4 sequence is 87,673 bp long and contains two segments of 5S rDNA: 5S–a and 5S–b (Fig. 1). The 5S rDNA fragments 5S–a and 5S–b were inserted into the third intron of G gene (positions 61,013 bp to 72,269 bp) and upstream of L gene (positions 82,474 bp to 87,673 bp) and contained 20 and 10 5S rDNA repeats, respectively. The 5S rDNA repeats in the BAC sequence were composed of a 120-bp CDS, 82-bp NTS, and 138-bp interposed region (IPR). Some of the IPRs contained A-repeats and TA-repeats. The longest and shortest 5S rDNA repeats in the BAC AT150B4 sequence were 964 bp and 201 bp, respectively. The 964-bp repeat contained five 138-bp IPRs between the 120-bp CDS and the 82-bp NTS.

### Heterozygosity of 4nAT

**Fig. 2:**
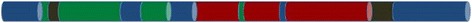
Genetic constitutions of the BAC clone 4nAT-150B4 full length sequence. The *red* color represents genomic DNA derived from RCC and the *green* color represents genomic DNA derived from CC. The variant DNA and conservative DNA are marked in *blue* and *black*, respectively. Variant DNA refers to DNA sequence that was absent in both parental genomes and conservative DNA refers to DNA sequence that was present in both genomes

By compared the BAC full length sequence 4nAT-150B4 with the genomes of RCC and CC, the characterization of genetic makeup of the BAC clone was obtained. The heterozygosity of 4nAT was confirmed by the genetic constitutions of 4nAT-150B4 BAC sequence (Fig. 2). In the BAC full length sequence, 34.29% of it was inherited from RCC genome, 27.14% of it was inherited from CC genome, 31.43% of it was variant DNA and 7.14% of it was conservative DNA.

### 5S rDNA organization in the four different fish species

**Fig. 3:**
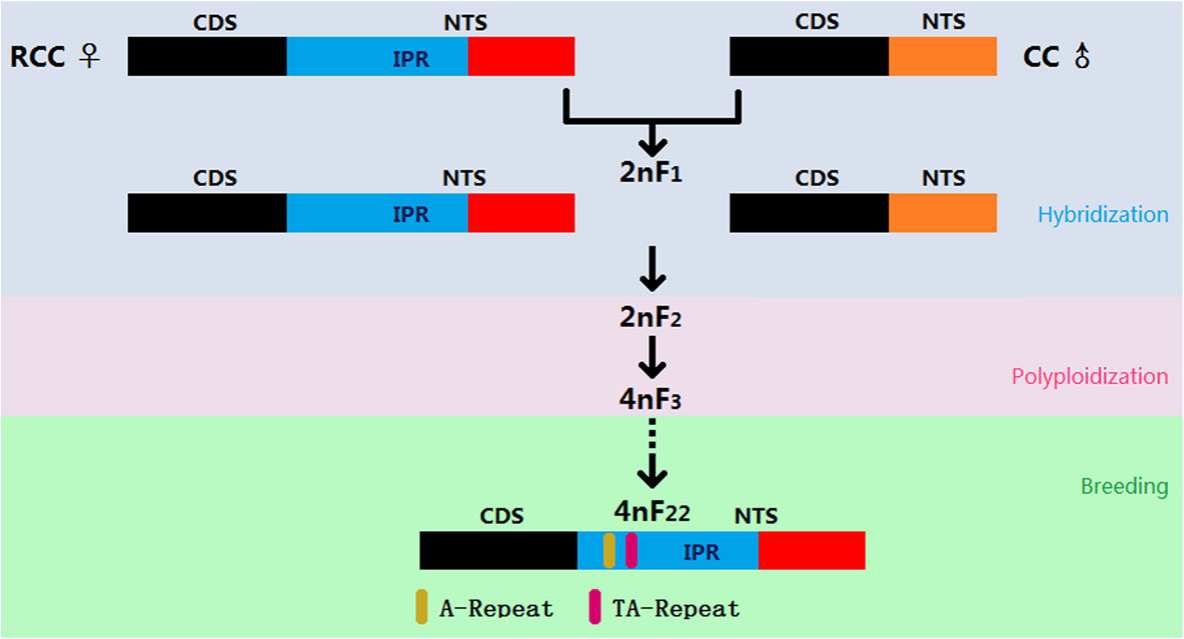
Organization of the 5S rDNA sequences detected in parental progenitors and hybrid offspring. The 82-bp NTS units in the 5S rDNA sequences in red crucian carp and common carp are marked in *red* and *orange*, respectively. The 138-bp IPRs are marked in *blue*. The three processes, hybridization, polyploidization, and breeding, are marked in different background colors. The highly similar regions in the 5S rDNA sequences of different fishes are marked in the same color

All the 5S rDNA sequences obtained by PCR and BLAST in the genomes of both progenitors, contained the 120-bp CDS and 82-bp NTS units (Fig. 3), and many of the 5S rDNA sequences in the maternal RCC contained varying numbers of 138-bp IPRs that did not contain the A-repeat and TA-repeat. The 5S rDNA sequences in the paternal CC did not contain 138-bp IPRs. The CDS regions of the 5S rDNAs in both progenitors were conserved, while the average similarity of the variable NTS regions between the two species was only 59.2%. The diploid hybrids 2nF_1_ inherited all the types of 5S rDNAs identified in both parents, and no new type of 5S rDNA was found in 2nF_1_. No A-repeat or TA-repeat were detected in the 138-bp IPR regions of the 5S rDNA sequences in 2nF_1_. Among the four fish species studied, the genetic variations of the 5S rDNA repeats were quite small in different individuals from the same species.

Most of the 5S rDNA repeats in BAC clone AT150B4 were composed of a CDS, NTS, and IPR, and only a small number of them did not contain an IPR. We obtained all of the 5S rDNAs detected in the BAC sequence by PCR, except for the 5S rDNA repeats that contained more than four 138-bp IPRs. A total of 72,138-bp IPR regions were detected in the BAC sequence and 29.2% and 8.3% of them contained an A-repeat or TA-repeat, respectively.

### Comparison of the 82-bp NTS units of 5S rDNA in the four different fish species

**Fig. 4:**
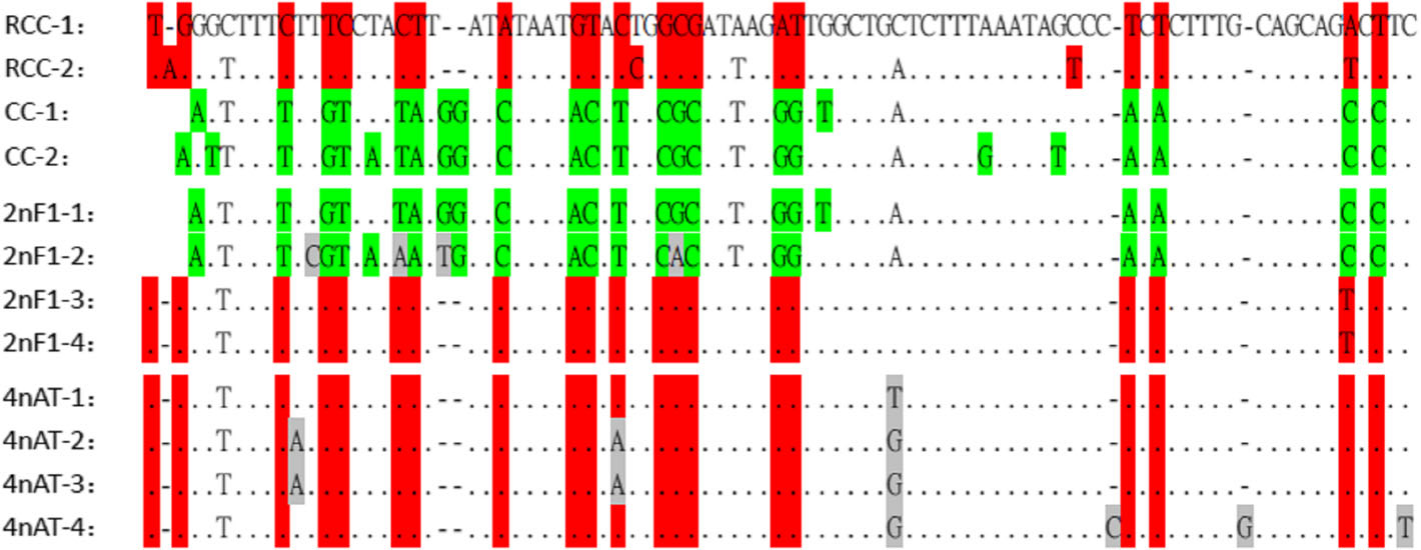
Multiple alignment of the 82-bp NTS units of 5S rDNAs in parental progenitors and hybrid progenies. Two representative 82-bp NTS units of both parental fishes and four representative 82-bp NTS units of both diploid hybrid 2nF_1_ and allotetraploid fish 4nF_22_ are shown. The nucleotide sites in *red* and *green* background are red crucian carp-specific locus and common carp-specific loci, respectively. The nucleotide sites in *gray* background are loci that vary in the hybrid progeny. The *dashes* represent gaps

The 82-bp NTS units of the 5S rDNA sequences in the four studied species were less conserved than the CDS regions. We detected 22 mutation sites between the 82- bp NTS sequences of 5S rDNA in the maternal and paternal progenitors (Fig. 4). The alignment shows that the first two 82-bp NTS units of 2nF_1_ were inherited from the paternal progenitor with four mutation sites in 2nF_1_–2, and the last two 82-bp NTS units of 2nF_1_ were inherited from the maternal progenitor (Fig. 4). All the 82-bp NTS units of 5S rDNA amplified from the 2nF_1_ genomic DNA that contained 138-bp IPRs were inherited from RCC, indicating that no homeologous recombination had occurred between the 5S rDNA sequences of the parental progenitors. For the 5S rDNA sequences obtained from BAC clone AT150B4 and amplified from the allotetraploid hybrid 4nAT, all of the 82-bp NTS units in 4nAT were inherited from maternal RCC and some mutational sites were detected (Fig. 4). The statistical analyses of the similarities of 82-bp NTS units between RCC and 2nF_1_, CC and 2nF_1_, RCC and 4nAT, and CC and 4nAT (Table 2) showed that the similarity of 82-bp NTS units between RCC and 2nF_1_ (mean 81.4%) was not statistically different than that between CC and 2nF_1_ (mean 79.3%) (u^a^ = 0.064 < u_0.05_ = 1.645). However, the similarity of 82-bp NTS units between RCC and 4nAT (mean 91.1%) was extremely significantly higher than that between CC and 4nAT (mean 62.0%) (u^b^ = 2.839 > u_0.01_ = 2.326).

**Table 2:** Statistical analyses of the 82-bp NTS units of 5S rDNA among different fish

Fish species	RCC	CC	
2nF_1_	(68.3–98.7)%, mean 81.4%	(53.7–100.0)%, mean 79.3%	u^a^ = 0.064
4nF_22_	(85.4–98.7)%, mean 91.1%	(52.4–72.0)%, mean 62.0%	u^b^ = 2.839

### Chromosomal locations of 5S rDNAs

**Fig. 5:**
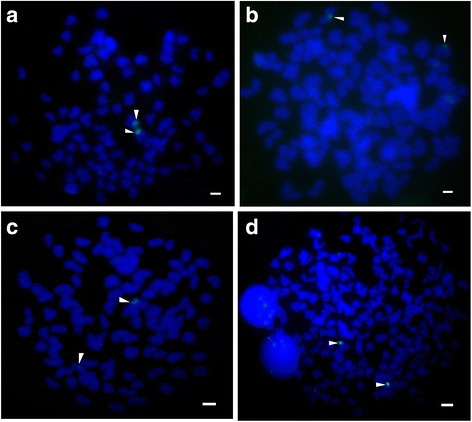
Location of 5S rDNA on chromosomes of hybrid fish 2nF_1_ and 4nAT and their parental species (detected by FISH). **a** Maternal red crucian carp. **b** Paternal common carp. **c** Diploid hybrid fish 2nF_1_. **d** Allotetraploid hybrid fish 4nAT. Scale bar = 3 μm

The above analyses have revealed that 200-bp 5S rDNA repeats emerged in the genomes of both progenitors, and diploid hybrid 2nF_1_ and tetraploid hybrid 4nAT. Therefore, we used the 200-bp 5S rDNA sequence amplified from paternal CC as a FISH probe to discover the chromosomal locations of the 5S rDNAs. We detected two 200-bp 5S rDNA signals in the chromosomes of each fish species (Fig. 5). The results indicated that the FISH signals of RCC chromosomes (Fig. 5a) were stronger than those of CC chromosomes (Fig. 5b). For the diploid hybrid fish 2nF_1_, a strong and a weak FISH signal were obtained, which indicated that one cluster of 5S rDNA was inherited from RCC, while the other was from CC (Fig. 5c). Unlike the 2nF_1_, the two FISH signals detected in allotetraploid hybrid fish 4nAT were both strong (Fig. 5d), which implied both of the 5S rDNA clusters were probably inherited from maternal RCC.

## Discussion

The difference between two types of 5S rDNAs relies mainly on the different features of the NTS unit, such as length and nucleotide variation. The NTS units seem to have been subject to rapid evolution. In the natural tobacco allotetraploids, *Nicotiana tabacum* [19] and *Nicotiana rustica* [20], two 5S rDNA families were identified that differed in the length of the NTS sequence. Similar differences in the NTS units have been described in 5S rDNAs from fishes such as *Oreochromis niloticus*, *Oncorhynchus mykiss*, *Coregonus* [21], *Merluccius* [10], and *Rhizoprionodon* sharks [22]. The nucleotide composition of the 82-bp NTS units varied in the 5S rDNAs from RCC and CC. Further, in RCC, some of the 5S rDNA sequences contained 138-bp IPRs, while the 5S rDNAs of CC had no IPRs. Therefore, we consider that the 5S rDNAs in the parental progenitors belong to two different classes.

In general, the genome of hybrid progeny would almost certainly mutate. For instance, evidence at a molecular level showed that chromosomal rearrangements took place rapidly in a newly formed diploid hybrid sunflower *Helianthus anomalus* [23]. However, as we stated above, two 5S rDNA classes, which were inherited from both progenitors, were found in diploid hybrid 2nF_1_. This result demonstrated that during the hybridization process of maternal RCC and paternal CC, the 5S rDNAs of the two progenitors were inherited independently, and showed an absence of homeologous recombination and interlocus gene conversion. However, a chimeric 5S rDNA has been observed in the genome of diploid and triploid hybrids of a female grass carp (*Ctenopharyngodon idellus*, Cyprinidae, 2n = 48) cross with male blunt snout bream (*Megalobrama amblycephala*, Cyprinidae, 2n = 48) [24]. Because the parents involved in the two cross combinations have identical chromosomal numbers, it cannot be concluded that the 5S rDNA sequences of the parents will certainly not be reconstructed in the process of distant hybridization in fish. However, it is reasonable that no signs of homeologous recombination were detected in the 5S rDNA sequence of diploid 2nF_1_ if the 5S rDNAs of the parental progenitors were distributed on non-homeologous chromosomes. Although, as suggested previously, intergenomic recombination was probably a major factor that contributes to genomic changes in newly synthesized hybrids [25].

Unlike the formation of allopolyploid plants [15, 25, 26], which is generally a result of distant hybridization, allotetraploid fish 4nAT were not generated from the distant hybridization of RCC and CC but from the fertilization of unreduced gametes produced by diploid 2nF_2_. Why there is this difference is still unknown. What is known, is the NTS units of 5S rDNAs in hybrid lineages have changed greatly after the process of tetraploidization. Our current results showed that the similarity of the 82-bp NTS units of 5S rDNA was significantly higher between 4nAT and RCC than between 4nAT and CC. In addition, the similarity of 82-bp NTS units of 5S rDNA in 4nAT and RCC showed no difference when compared with that of different 82-bp NTS units of 5S rDNA in 4nAT. It can be supposed that the CC-type 82-bp NTS units of 5S rDNAs detected in diploid 2nF_1_ might no longer existed in tetraploid hybrids. However, because the genome of 4nAT is complicated, and the sample in this research was limited, so we cannot absolutely concluded that all of the 4nAT individuals do not contain CC-type 82-bp NTS units. It is important to point out that the hybridity of 4nAT was validated by tangible evidence [27]. We also noticed that the 5S rDNAs of 4nAT not only contained 138-bp IPRs (unlike the 5S rDNAs of RCC, the 5S rDNAs of CC did not contain 138-bp IPRs), but the 138-bp IPRs contained A-repeats and TA-repeats. Considering slipped-strand mispairing is responsible for the evolution of tandem repeats, we deduced that the insertion of A-repeats and TA-repeats may be the result of slipped-strand mispairing during the polyploidization. Two strong FISH signals were detected in the tetraploid hybrid chromosomes, as well as in the maternal progenitor RCC, while one strong and one weak signals were detected in the diploid hybrid chromosomes. In allopolyploid tobacco, the 5S rDNA structural organization characteristics of the parental species have generally been preserved [7]. Similarly, interlocus homogenization at 5S rDNA has not been observed in allopolyploid *Gossypium* species [28]. In the synthetic allopolyploid *Triticum* × *Aegilops*, little or no change of parental 5S rDNA repeats was observed; thus, interlocus gene conversion and interlocus homogenization had not occurred for the 5S rDNAs [15]. Why the 5S rDNAs of the tetraploid hybrid fish species in this study were different from the 5S rDNAs of polyploid plants may be explained as follows. The allopolyploid plants were generated passively from distant hybridization, while the tetraploidization of the hybrid lineage in this research was an active process quite independent of distant hybridization. Certainly, the interaction rate of sequences share a certain level of homology would increase when the chromosome number of hybrid fish doubled to 200. Biased gene conversion of 5S rDNAs in the hybrid lineage seems to have occurred during the process of tetraploidization.

## Conclusions

It is notable that the NTS units of 5S rDNAs in the parental red crucian carp and common carp species were different from each other. After a comprehensive analysis of 5S rDNAs in diploid 2nF_1_, we found the 5S rDNAs of both parental progenitors were inherited stably during the process of hybridization. However, tandem repeat insertion events occurred in the allotetraploid fish during the process of polyploidization. Additionally, the 5S rDNAs in the allotetraploid fish probably underwent an interlocus gene conversion event along with tetraploidization based on our current data. Admittedly, hard evidence is still lacking to explain why this was the case. However, our findings will be very valuable for improving our knowledge about how hybridization and polyploidization can affect the evolution of genes in hybrids.

## Abbreviations

2nF_1_: the first filial generation of red crucian carp × common carp
4nAT: allotetraploid hybrid fish
4nF_22_: the 22nd-generation tetraploid hybrids of red crucian carp × common carp
BAC: Bacterial artificial chromosome
BES: BAC-ends
CC: Common carp
CDS: Coding sequence
FISH: Fluorescence in situ hybridization
IPRs: Interposed regions
NTS: Non-transcribed spacer
RCC: Red crucian carp

## References

[1] Xu P, Zhang X, Wang X, Li J, Liu G, Kuang Y, Xu J, Zheng X, Ren L, Wang G, Zhang Y, Huo L, Zhao Z, Cao D, Lu C, Li C, Zhou Y, Liu Z, Fan Z, Shan G, Li X, Wu S, Song L, Hou G, Jiang Y, Jeney Z, Yu D, Wang L, Shao C, Song L, Sun J, Ji P, Wang J, Li Q, Xu L, Sun F, Feng J, Wang C, Wang S, Wang B, Li Y, Zhu Y, Xue W, Zhao L, Wang J, Gu Y, Lv W, Wu K, Xiao J, Wu J, Zhang Z, Yu J, Sun X (2014). Genome sequence and genetic diversity of the common carp, *Cyprinus carpio*. Nat Genet.

[2] Liu S, Liu Y, Zhou G, Zhang X, Luo C, Feng H, He X, Zhu G, Yang H (2001). The formation of tetraploid stocks of red crucian carp × common carp hybrids as an effect of interspecific hybridization. Aquaculture.

[3] Long EB, David IB (1980). Repeated genes in eukaryotes. Annu Rev Biochem.

[4] Keller I, Chintauan-Marquier IC, Veltsos P, Nichols RA (2006). Ribosomal DNA in the grasshopper Podisma pedestris: escape from concerted evolution. Genetics.

[5] Freire R, Arias A, Insua A, Méndez J, Eirín-López JM (2010). Evolutionary dynamics of the 5S rDNA gene family in the mussel Mytilus: mixed effects of birth-and-death and concerted evolution. J Mol Evol.

[6] Rooney AP, Ward TJ (2005). Evolution of large ribosomal RNA multigene family in filamentous fungi: birth and death of a concerted evolution paradigm. PNAS.

[7] Fulnecek J, Lim KY, Leitch AR, Kovarík A, Matyásek R (2002). Evolution and structure of 5S rDNA loci in allotetraploid Nicotiana tabacum and its putative parental species. Heredity.

[8] Merlo MA, Cross I, Manchado M, Cárdenas S, Rebordinos L (2013). The 5S rDNA high dynamism in *Diplodus sargus* is a transposon-mediated mechanism. Comparison with other multigene families and Sparidae species. J Mol Evol.

[9] Pinhal D, Yoshimura TS, Araki CS, Martins C (2011). The 5S rDNA family evolves through concerted and birth-and-death evolution in fish genomes: an example from freshwater stingrays. BMC Evol Biol.

[10] Campo D, Machado-Schiaffino G, Horreo JL, Garcia-Vazquez E (2009). Molecular organization and evolution of 5S rDNA in the genus *Merluccius* and their phylogenetic implications. J Mol Evol.

[11] Merlo MA, Pacchiarini T, Portela-Bens S, Cross I, Manchado M, Rebordinos L (2012). Genetic characterization of *Plectorhinchus mediterraneus* yields important clues about genome organization and evolution of multigene families. BMC Genet.

[12] Drouin G, de Sá MM (1995). The concerted evolution of 5S ribosomal genes linked to the repeat units of other multigene families. Mol Biol Evol.

[13] Boroń A, Ozouf-Costaz C, Coutanceau JP, Woroniecka K (2006). Gene mapping of 28S and 5S rDNA sites in the spined loach *Cobitis taenia* (Pisces, Cobitidae) from a diploid population and a diploid-tetraploid population. Genetica.

[14] Martins C, Galetti PM (1999). Chromosomal localization of 5S rDNA genes in Leporinus fish (Anostomidae, Characiformes). Chromosom Res.

[15] Shcherban’ AB, Sergeeva EM, Badaeva ED, Salina EA (2008). Analysis of 5S rDNA changes in synthetic allopolyploids *Triticum* × *Aegilops*. Mol Biol.

[16] Wang J, Ye LH, Liu QZ, Peng LY, Liu W, Yi XG, Wang YD, Xiao J, Xu K, Hu FZ, Ren L, Tao M, Zhang C, Liu Y, Hong YH, Liu SJ (2015). Rapid genomic DNA changes in allotetraploid fish hybrids. Heredity.

[17] Masaru M, Hideo F (1998). Characterization of repetitive DNA sequences carrying 5S rDNA of the triploid *ginbuna*. Genes Genet Syst.

[18] Zhang C, Ye LH, Chen Y, Xiao J, Wu YH, Tao M, Xiao Y, Liu S (2015). The chromosomal constitution of fish hybrid lineage revealed by 5S rDNA FISH. BMC Genet.

[19] Fulnecek J, Matyásek R, Kovarík A, Bezdek M (1998). Mapping of 5-methylcytosine residues in Nicotiana tabacum 5S rRNA genes by genomic sequencing. Mol Gen Genet.

[20] Venkateswarlu K, Lee SW, Nazar RN (1991). Conserved upstream sequence elements in plant 5S ribosomal RNAencoding genes. Gene.

[21] Martins C, Galetti PM (2001). Two 5S rDNA arrays in Neotropical fish species: is it a general rule for fishes?. Genetica.

[22] Pinhal D, Araki CS, Gadig OB, Martins C (2009). Molecular organization of 5S rDNA in sharks of the genus *Rhizoprionodon*: insights into the evolutionary dynamics of 5S rDNA in vertebrate genomes. Genet Res (Camb).

[23] Rieseberg LH, Sinervo B, Linder CR, Ungerer MC, Arias DM (1996). Role of gene interactions in hybrid speciation: evidence from ancient and experimental hybrids. Science.

[24] He WG, Xie LH, Li TL, Liu SJ, Xiao J, Hu J, Wang J, Qin Q, Liu Y (2013). The formation of diploid and triploid hybrids of female grass carp × male blunt snout bream and their 5s rDNA analysis. BMC Genet.

[25] Song K, Lu P, Tang K, Osborn TC (1995). Rapid genome change in synthetic polyploids of *Brassica* and its implications for polyploid evolution. PNAS.

[26] Tate JA, Symonds VV, Doust AN, Buggs RJ, Mavrodiev E, Majure LC, Soltis PS, Soltis DE (2009). Synthetic polyploids of *Tragopogon miscellus* and *T. mirus* (Asteraceae): 60 Years after Ownbey's discovery. Am J Bot.

[27] Liu S, Luo J, Chai J, Ren L, Zhou Y, Huang F, Liu X, Chen Y, Zhang C, Tao M, Lu B, Zhou W, Lin G, Mai C, Yuan S, Wang J, Li T, Qin Q, Feng H, Luo K, Xiao J, Zhong H, Zhao R, Duan W, Song Z, Wang Y, Wang J, Zhong L, Wang L, Ding Z, Du Z, Lu X, Gao Y, Murphy RW, Liu Y, Meyer A, Zhang Y-P (2016). Genomic incompatibilities in the diploid and tetraploid offspring of the goldfish × common carp cross. PNAS.

[28] Cronn RC, Zhao X, Paterson AH, Wendel JF (1996). Polymorphism and concerted evolution in a tandemly repeated gene family: 5S ribosomal DNA in diploid and allopolyploid cottons. J Mol Evol.

